# Chronic Calcific Pancreatitis as the Probable Initial Manifestation of Systemic Lupus Erythematosus With Mixed Connective Tissue Disease Overlap: A Case Report

**DOI:** 10.1002/ccr3.73909

**Published:** 2026-11-09

**Authors:** Rashid Shahriar Sazal, Abdullah Al Faisal, Sabiha Sikder, Moontasir Ahmed, Md. Riyaz Uddin Sk. Mubin, Sakin Saleh Ifti

**Affiliations:** ^1^ Department of Internal Medicine Bangladesh Medical University Dhaka Bangladesh; ^2^ Department of Internal Medicine Dhaka National Medical College Dhaka Bangladesh; ^3^ Department of Internal Medicine Tangail Medical College Hospital Tangail Bangladesh; ^4^ Department of Internal Medicine Shaheed Suhrawardy Medical College Hospital Dhaka Bangladesh

**Keywords:** chronic calcific pancreatitis, inflammatory myositis, mixed connective tissue disease, overlap syndrome, systemic lupus erythematosus

## Abstract

Chronic calcific pancreatitis as the presenting manifestation of systemic lupus erythematosus (SLE) is exceedingly rare, particularly in tropical regions where it may be misattributed to tropical chronic pancreatitis (TCP). A 22‐year‐old Bangladeshi male farmer presented with a one‐year history of recurrent postprandial epigastric pain and steatorrhea. Three months before admission, he developed inflammatory polyarthritis, a photosensitive malar rash, oral ulcers, Raynaud phenomenon, and progressive proximal muscle weakness. Investigations revealed normocytic anemia (hemoglobin 9.2 g/dL), thrombocytopenia (50 × 10^9^/L), a positive direct Coombs test, and a broad autoantibody profile including strongly positive antinuclear antibodies, anti‐dsDNA, anti‐U1‐RNP, anti‐nucleosome, anti‐Ku, and anti‐ribosomal P antibodies. Computed tomography and magnetic resonance cholangiopancreatography showed chronic calcific pancreatitis with beaded main pancreatic duct dilatation (10–11 mm), diffuse intraductal calculi, and parenchymal atrophy, without features of autoimmune pancreatitis. He fulfilled the 2019 EULAR/ACR classification criteria for SLE (score 22) and the Sharp criteria for mixed connective tissue disease (MCTD) overlap. Alcohol use, gallstone disease, hypercalcemia, hypertriglyceridemia, pancreatotoxic drugs, and cassava exposure were absent, but genetic testing and serum IgG4 were unavailable. Hydroxychloroquine, prednisolone, mycophenolate mofetil, and pancreatic enzyme replacement were followed by improvement of the systemic features, including platelet normalization. On the available evidence, the pancreatic disease is most plausibly an early manifestation of SLE–MCTD overlap, although a hereditary or tropical contribution cannot be formally excluded. This case expands the spectrum of SLE‐associated pancreatic disease and underscores the need for systematic autoimmune evaluation in young patients from tropical regions with unexplained chronic calcific pancreatitis.

## Key Clinical Message

Chronic calcific pancreatitis may be a presenting manifestation of SLE with MCTD overlap, preceding the systemic features. In young patients from tropical regions with unexplained pancreatic calcification, a comprehensive autoimmune evaluation, alongside hereditary and tropical work‐up where available, can avoid misdiagnosis as tropical chronic pancreatitis and enable timely immunosuppression.

## 1 Introduction

Systemic lupus erythematosus (SLE) is a protean autoimmune disorder in which clinically significant pancreatic involvement is estimated at 0.7%–4.2% of patients, predominantly as acute pancreatitis [1, 2, 3, 4]. Chronic pancreatitis in SLE is much rarer and is described mainly in isolated case reports [5, 6, 7]; it is usually recognized years after SLE onset. The pancreas may nonetheless be the first organ to declare itself, because autoantibodies can precede clinical SLE by years [8]. Mixed connective tissue disease (MCTD), characterized by high‐titre anti‐U1‐RNP antibodies with overlapping features of SLE, polymyositis, and systemic sclerosis, may further complicate the clinical picture [9, 10]. In young patients from tropical agricultural regions, chronic calcific pancreatitis is more often attributed to tropical or hereditary causes, and an autoimmune cause may be overlooked. We report a young South Asian man in whom chronic calcific pancreatitis preceded multisystem autoimmunity by about 9 months and was followed by confirmed SLE with MCTD overlap and anti‐Ku positivity, and we discuss the diagnostic, mechanistic, and therapeutic dimensions of this association.

## 2 Case Presentation

### 2.1 Patient Information

A 22‐year‐old Bangladeshi male farmer with no prior medical or surgical history and no documented hereditary risk factors presented with a 1‐year history of recurrent postprandial epigastric pain radiating to the back and intermittent steatorrhea. Three months before admission he developed symmetrical inflammatory polyarthritis of the small joints, a photosensitive malar rash, recurrent oral ulcers (as shown in Figure 1), Raynaud phenomenon of the fingers, and progressive proximal muscle weakness. There was no alcohol use, no gallstone disease, no abdominal trauma, no exposure to cassava or other cyanogenic‐glycoside–rich foods, and no history of pancreatotoxic medication (including azathioprine, valproate, or diuretics).

**FIGURE 1:**
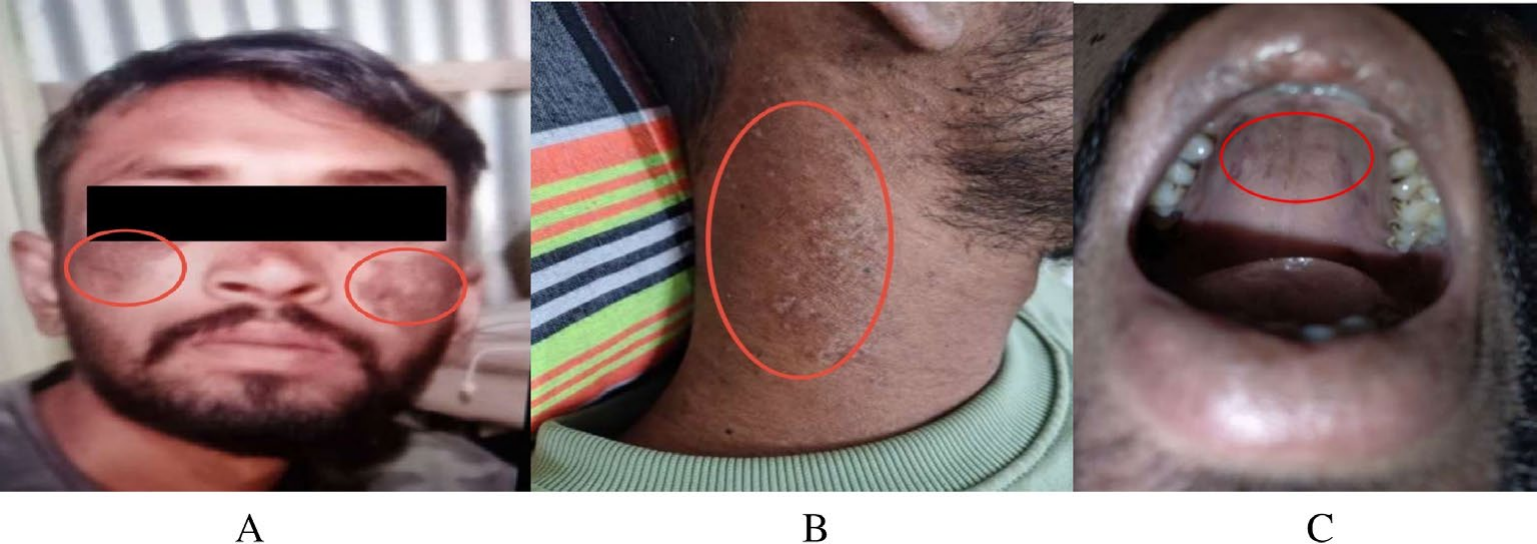
Clinical photographs of the patient showing well‐demarcated hyperpigmented macules and plaques over the malar region and cheek, sparing the nasolabial folds, butterfly distribution (1A); lateral view of the right side of the neck showing clustered, hyperpigmented plaque‐like lesions with mild surface texturing (1B); multiple erythematous, irregular shallow ulcerated lesions over the hard palate (1C).

### 2.2 Clinical Findings

Examination revealed pallor, a well‐demarcated malar rash sparing the nasolabial folds, hyperpigmented plaques over the lateral neck (Figure 1), hard‐palate ulceration, proximal interphalangeal synovitis, symmetrical proximal muscle weakness (Medical Research Council grade 3/5 in lower limbs), a positive Gower sign, and mild splenomegaly. Vital signs and fundoscopy were unremarkable; neuropsychiatric features were absent.

### 2.3 Diagnostic Assessment

Laboratory evaluation showed normocytic anemia (Hb 9.2 g/dL), thrombocytopenia (50 × 10^9^/L), elevated ESR (83 mm/h) and CRP (12.5 mg/dL), mildly elevated CPK (312 U/L; normal ≤ 170 U/L), positive direct Coombs test, normal renal and hepatic biochemistry, normal serum amylase and lipase, and normal calcium, triglycerides, and complement. Autoantibody profiling showed ANA 352 AU/mL, anti‐dsDNA 201.26 U/mL, anti‐U1‐RNP, anti‐nucleosome, anti‐Ku, and anti‐ribosomal P positivity; rheumatoid factor and anti‐CCP were negative. Antiphospholipid testing (anti‐cardiolipin, anti‐β2‐glycoprotein I, lupus anticoagulant) was negative. Plain radiograph of the abdomen showed multiple irregular calcified shadows at the L2–L3 level consistent with pancreatic calculi (Figure 2). Non‐contrast abdominal CT demonstrated numerous radio‐dense structures throughout the pancreas and within a dilated main pancreatic duct (11 mm), with the largest calculus measuring ~26 × 13 mm near the pancreatic tail and accompanying parenchymal atrophy; the liver was mildly enlarged (Figure 3). MRCP confirmed beaded main pancreatic duct dilatation (10 mm), diffuse intraductal ductolithiasis, and parenchymal atrophy, without the “sausage‐shaped” pancreas, peripancreatic halo, or delayed‐enhancement pattern of type 1 autoimmune pancreatitis, and without biliary abnormality. Genetic testing for hereditary pancreatitis (PRSS1, SPINK1, CFTR, CTRC) was not performed owing to resource constraints, and serum IgG4 was not available at our facility. No hereditary risk factors were documented, but a hereditary contribution cannot be formally excluded. The patient fulfilled the 2019 EULAR/ACR SLE classification criteria with a weighted score of 22 (entry criterion: ANA ≥ 1:80; clinical domains scored: constitutional, hematologic, mucocutaneous, musculoskeletal; threshold ≥ 10) [11] and Sharp criteria for MCTD overlap [12]. The clinical timeline is summarized in Table 1.

**FIGURE 2:**
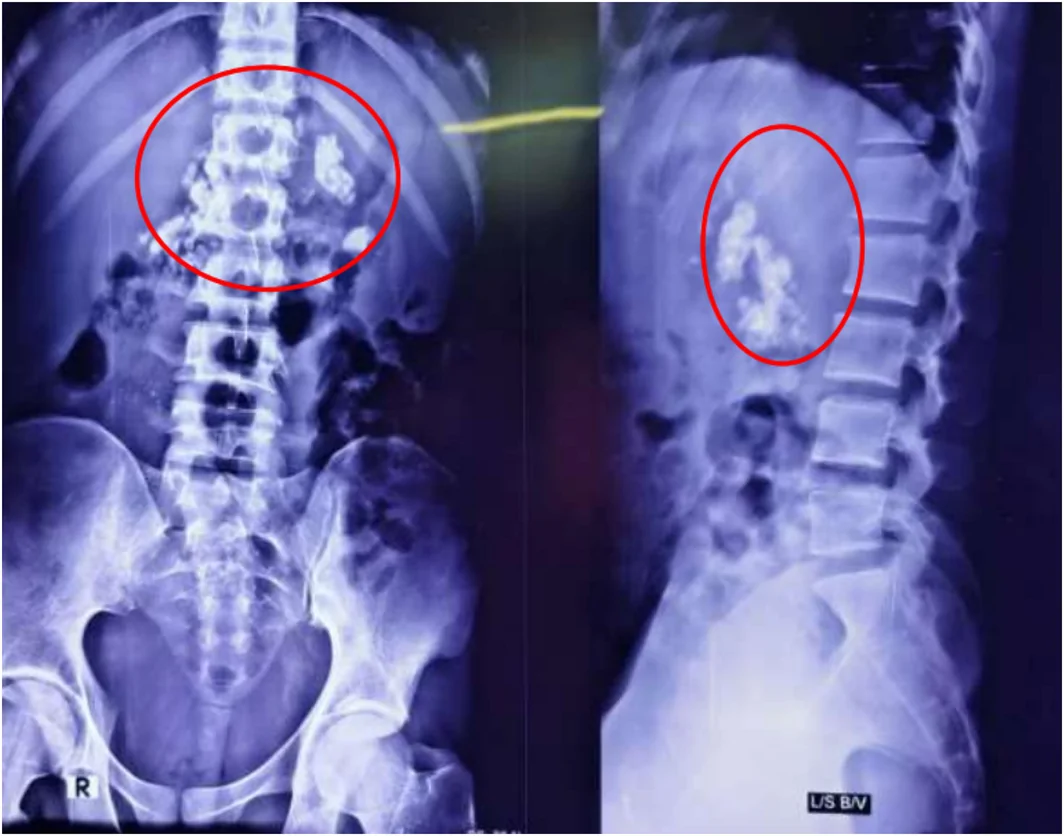
Plain X‐Ray abdomen, antero‐posterior, and lateral view in erect posture showing multiple irregular calcified shadows in the upper abdomen at the level of L2 and L3, suggestive of pancreatic calculi.

**FIGURE 3:**
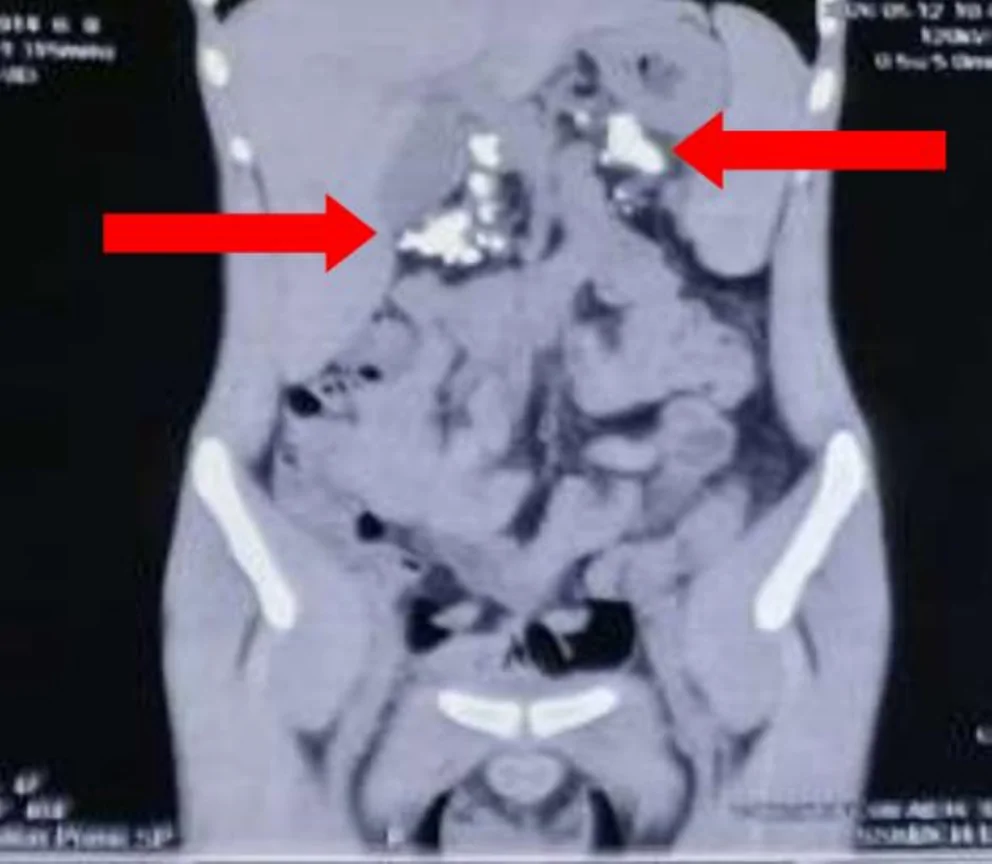
Non‐Contrast CT scan of abdomen showing numerous radiodense structures throughout the pancreas and within dilated main pancreatic duct, the largest one measuring about 26 × 13 mm near the tail. Liver is mildly enlarged in size with uniform density.

**TABLE 1:** Timeline of clinical course, investigations, and management.

Timeline	Clinical findings/investigations	Management/outcome
12 months pre‐admission	Recurrent postprandial epigastric pain, steatorrhea.	Symptomatic analgesia only.
3 months pre‐admission	Inflammatory polyarthritis, malar rash, oral ulcers, Raynaud phenomenon, proximal myopathy.	No treatment initiated.
Day 1 (Admission)	Hb 9.2 g/dL; platelets 50 × 10^9^/L; ESR 83 mm/h; CRP 12.5 mg/dL; CPK 312 U/L; Coombs+. XR: pancreatic calcifications. Renal/hepatic function, amylase, lipase: normal.	Analgesia, nutritional support, pancreatin initiated.
Days 2, 3	USS: pancreatic calculi, MPD dilatation. ANA 352 AU/mL; anti‐dsDNA 201.26 U/mL.	HCQ 300 mg/day; prednisolone 60 mg/day commenced.
Day 4	ENA: anti‐U1‐RNP, anti‐nucleosome, anti‐Ku, anti‐RPP/PO positive. 2019 EULAR/ACR SLE criteria fulfilled (score 22). MCTD overlap confirmed.	Diagnosis established.
Day 5	CT abdomen: chronic calcific pancreatitis, beaded MPD (11 mm), parenchymal atrophy, hepatosplenomegaly. No AIP features.	Surgical consultation; conservative management continued.
Day 7	MRCP: MPD 10 mm, intraductal calculi, ductolithiasis confirmed. No biliary abnormality.	Prednisolone tapered to 40 mg/day; MMF 500 mg/day added.
Day 9 (Discharge)	Platelets normalized (50 → 155 × 10^9^/L). Clinical improvement across all domains.	Discharged on HCQ, tapering prednisolone, MMF, pancreatin, nutritional support.
Follow‐up	Sustained improvement in pain, arthritis, muscle strength. No recurrence.	Joint rheumatology/gastroenterology surveillance ongoing.

Other common causes of chronic calcific pancreatitis were sought and not found: there was no alcohol use, gallstone disease, or biliary abnormality on MRCP, hypercalcemia, hypertriglyceridemia, abdominal trauma, or pancreatotoxic medication. Differential diagnoses included tropical chronic pancreatitis (considered unlikely given the absence of cassava exposure, the normal nutritional biomarkers, and the evolving multisystem autoimmunity, although it cannot be formally excluded without genetic testing), autoimmune pancreatitis type 1 (not supported by the imaging morphology, although serum IgG4 was unavailable), and seronegative rheumatoid arthritis with extra‐articular involvement (excluded by dominant SLE serology).

### 2.4 Therapeutic Intervention

Initial management consisted of oral pancreatin, analgesia (avoiding NSAIDs owing to thrombocytopenia), and nutritional support. Hydroxychloroquine 300 mg/day and prednisolone 60 mg/day were started on Days 2–3, once the clinical and serological picture indicated active SLE; prednisolone was tapered to 40 mg/day by Day 7, when mycophenolate mofetil 500 mg/day was added as a steroid‐sparing agent.

### 2.5 Follow‐Up and Outcomes

Platelet count normalized by Day 9 of admission (50 to 155 × 10^9^/L). Symptomatic improvement across all disease domains was documented: reduced arthralgia, resolution of oral ulcers, and improvement in proximal muscle strength from MRC 3/5 to 4/5. At follow‐up, the improvement in abdominal pain, arthritis, and muscle strength was sustained, with no recurrence of symptoms. Because pancreatic enzyme replacement and supportive care were started together with immunosuppression, the improvement in pancreatic symptoms cannot be attributed to immunosuppression alone, and the calcific ductal changes are structural and not expected to regress. Improvement attributable to immunosuppression was evident in the extra‐pancreatic domains, including platelet normalization, arthritis, oral ulceration, and muscle strength. Multidisciplinary rheumatology–gastroenterology surveillance is continuing with SLE disease‐activity tracking (SLEDAI‐2 K), serial anti‐dsDNA and CPK monitoring, periodic neuropsychiatric symptom screening [13], baseline and periodic high‐resolution chest CT in view of the documented anti‐Ku‐related interstitial‐lung‐disease risk [14, 15], and structured pancreatic endocrine (HbA1c, fasting glucose) and exocrine (fecal elastase‐1, nutritional status) surveillance.

## 3 Discussion

This case documents chronic calcific pancreatitis as the presenting manifestation of SLE–MCTD overlap [16], preceding the systemic features by about 9 months. Pancreatic involvement is described in 0.7%–4.2% of patients with SLE and presents predominantly as acute pancreatitis [1, 2, 3, 4]. Chronic pancreatitis is much less common: in the Hopkins Lupus Cohort, 9 of 63 patients (14%) with SLE‐related pancreatitis developed chronic disease [4], and chronic steroid use may mask abdominal pain and delay recognition [17]. Isolated reports describe chronic calcifying pancreatitis in patients with SLE after common causes were sought and not found [5, 6, 7].

Our case fits this literature in two respects. First, the pancreatic disease and the autoimmune disease appeared in close sequence in a young patient without the usual risk factors. Second, calcific change can evolve quickly in SLE: in the patient described by Gutierrez et al., the first episode of abdominal pain occurred at the onset of SLE and chronic calcifying pancreatitis was established 2 years later [7]. Chronic pancreatitis is usually diagnosed years after the SLE diagnosis, but that describes when it was recognized, not necessarily when it began. Autoantibodies can precede clinical SLE by years [8], so an immune‐mediated pancreatic process could plausibly have been the first clinical expression of an evolving autoimmune disease. In our patient, the pancreatic symptoms preceded the systemic features by about 9 months, and large intraductal calculi (up to 26 × 13 mm) and glandular atrophy were already present at presentation, in keeping with ductal injury of long standing that predated clinical recognition of the autoimmune disease.

The proposed mechanisms of pancreatic injury in SLE include immune‐complex deposition within pancreatic vasculature, complement‐mediated acinar cell injury, small‐vessel vasculitis, and antiphospholipid antibody‐driven microvascular thrombosis [3, 4]. Antiphospholipid antibodies were negative in our patient, so immune‐complex or vasculitic injury is the likelier mechanism, although no pancreatic tissue was available to test this. The broad, strongly positive autoantibody profile, the multisystem activity at presentation, and the improvement of the systemic features with immunosuppression indicate active autoimmune disease when the pancreatic disease was recognized.

The South Asian tropical context adds a diagnostic pitfall in both directions. Tropical chronic pancreatitis, linked to cassava toxicity, malnutrition, and SPINK1 variants, characteristically affects young individuals from agricultural communities [18, 19], a profile that mirrors our patient and creates a risk of anchoring bias, in which pancreatic calcification is reflexively attributed to a tropical cause and the autoimmune evaluation is foreclosed. Against tropical chronic pancreatitis in our patient were the absence of cassava exposure, the normal nutritional biomarkers, and the evolving multisystem autoimmunity. A hereditary contribution cannot be formally excluded because PRSS1, SPINK1, CFTR, and CTRC testing was not possible, although no hereditary risk factors were documented. SPINK1 variants may require a second hit to manifest, and active SLE has been reported as such a trigger [20], although that patient had acute necrotizing pancreatitis without pancreatic calcification. Testing for these genes is therefore advisable in similar patients where it is available. On the available evidence, we regard SLE–MCTD overlap as the most plausible explanation of the pancreatic disease in this patient, while acknowledging that a coexisting hereditary or tropical predisposition cannot be formally excluded and that these possibilities are not mutually exclusive.

The response to treatment needs cautious interpretation. Immunosuppression was followed by clear improvement of the extra‐pancreatic disease, including platelet normalization, arthritis, oral ulceration, and muscle strength. Improvement in pancreatic symptoms cannot be attributed to immunosuppression alone, because pancreatic enzyme replacement and supportive care were started together with it, and calcified ductal stones are structural lesions that are not expected to regress. Whether early immunosuppression can modify the course of SLE‐related pancreatic disease before extensive calcification is unknown. The subsequent pancreatic course will be informative: progression of pancreatic disease while SLE is controlled would favor an independent process, whereas pancreatic flares that parallel SLE activity and settle with immunosuppression would favor an association.

The autoantibody profile reflects poly‐autoimmunity, the co‐occurrence of multiple autoimmune phenotypes driven by shared immunological susceptibilities [21]. Anti‐dsDNA and anti‐nucleosome antibodies carry high SLE specificity and mediate immune complex‐driven organ vasculopathy [22]. Anti‐U1‐RNP antibodies define the MCTD overlap and associate with pulmonary hypertension and inflammatory myositis [10, 23]. Anti‐Ku antibodies, markers of polymyositis–SLE overlap, confer risk of myositis [24], Raynaud phenomenon, and interstitial lung disease; myositis and Raynaud phenomenon were present in our patient [25, 26]. Anti‐ribosomal P antibodies are classically linked to neuropsychiatric and myopathic lupus [26]. Neuropsychiatric involvement is an important extra‐renal manifestation of SLE and may emerge later in the disease course or at relapse. One report described a young woman with childhood‐onset SLE and prior lupus nephritis who presented with bilateral upward gaze palsy and intraoral numbness from an inflammatory midbrain lesion; antiphospholipid antibodies were absent, and the gaze palsy and MRI lesion resolved with intensive immunosuppression [13]. Although our patient has no neuropsychiatric features to date, his anti‐ribosomal P positivity supports periodic neurological review and a low threshold for brain MRI if symptoms arise. The myopathic component (proximal weakness, MRC 3/5 in the lower limbs; positive Gower sign; and elevated creatine phosphokinase) is consistent with lupus myositis or overlap myositis in the MCTD context. Formal electromyography and muscle biopsy remain planned per the 2017 EULAR/ACR criteria for idiopathic inflammatory myopathies [27].

The combination of hydroxychloroquine, prednisolone, and mycophenolate mofetil was followed by rapid improvement of the systemic features, supporting early immunosuppression for the autoimmune component. Corticosteroid responsiveness of the myopathic component is consistent with the established anti‐Ku muscle‐disease pattern, in which complete muscle remission has been achieved with steroids in roughly 73% of patients with inflammatory myopathy [15]. However, the same cohort experience shows lung disease being cortico‐resistant in about 75% of treated cases, a finding that mandates baseline high‐resolution chest CT and longitudinal pulmonary function surveillance even in the absence of respiratory symptoms [14, 15]. Ongoing pancreatic surveillance is essential given the documented ductolithiasis and the progressive risk of endocrine and exocrine insufficiency.

## 4 Strengths and Limitations

This case is strengthened by a comprehensive autoantibody profile alongside fulfillment of two validated classification criteria, which provide robust confirmation of the autoimmune diagnosis. The common causes of chronic calcific pancreatitis (alcohol, gallstone and biliary disease, hypercalcemia, hypertriglyceridemia, medications, and trauma) were documented as absent, and multimodal pancreatic imaging documented the chronicity and structural severity of the pancreatic disease. Genetic testing for hereditary pancreatitis (PRSS1, SPINK1, CFTR, CTRC) and serum IgG4 measurement were not possible because of resource constraints, and no pancreatic tissue was obtained. Hereditary and other structural causes of chronic calcific pancreatitis therefore cannot be formally excluded, and the proposed relationship between SLE and the pancreatic disease remains an inference from a single case. Formal electromyography and muscle biopsy were not performed, precluding histopathological classification of the myopathic component. Long‐term follow‐up data remain limited to the early post‐discharge period.

## 5 Conclusion

Chronic calcific pancreatitis can be a presenting manifestation of SLE–MCTD overlap and, on the available evidence, most plausibly was in this young man, although a hereditary or tropical contribution could not be formally excluded. In young patients from tropical regions, reflexive attribution to tropical chronic pancreatitis risks diagnostic delay; systematic autoimmune evaluation, including ANA, anti‐dsDNA, and ENA profile, is imperative alongside hereditary work‐up where available. Early combination immunosuppression, together with pancreatic‐directed management, was followed by improvement of the systemic features in this patient. Multidisciplinary care involving rheumatology, gastroenterology, and hepatopancreatobiliary surgery is essential.

### 5.1 Patients' Perspective

The patient described significant distress from recurrent abdominal pain, weight loss, joint pain and progressive weakness, which interfered with his daily farming activities and quality of life. Before diagnosis, he believed his illness was limited to a stomach problem and did not anticipate any autoimmune disease. Following treatment, he experienced marked improvement in his symptoms and overall functional capacity. He expressed gratitude for the multidisciplinary care and agreed to share his clinical experience to improve awareness of such rare diseases.

## Author Contributions

**Rashid Shahriar Sazal:** conceptualization, data curation, writing – original draft, investigation, methodology. **Abdullah Al Faisal:** writing – review and editing, writing – original draft, visualization, methodology, data curation. **Sabiha Sikder:** writing – review and editing, investigation, resources, software, methodology. **Moontasir Ahmed:** writing – review and editing, software, resources, validation, formal analysis. **Md. Riyaz Uddin Sk. Mubin:** writing – review and editing, formal analysis, resources, software, visualization. **Sakin Saleh Ifti:** writing – review and editing, project administration, supervision, funding acquisition, validation.

## Funding

The authors have nothing to report.

## Ethics Statement

According to institutional case report regulations, this report was cleared from formal ethical review.

## Consent

Written informed consent was given by the patient along with accompanying images. A copy of the written consent is available for review by the Editor‐in‐chief of this journal.

## Conflicts of Interest

The authors declare no conflicts of interest.

## Data Availability

The data generated or analyzed during this case report are included in this published article. Additional data are available from the corresponding author upon reasonable request, subject to patient confidentiality.
